# Numerical Simulation and Analytical Prediction of Residual Strength for Elbow Pipes with Erosion Defects

**DOI:** 10.3390/ma15217479

**Published:** 2022-10-25

**Authors:** Chao Sun, Qi Wang, Yuelin Li, Yingqi Li, Yuechan Liu

**Affiliations:** School of Measurement and Communication Engineering, Harbin University of Science and Technology, Harbin 150080, China

**Keywords:** elbow pipes, analytical prediction, residual strength, failure pressure, erosion defects

## Abstract

It is well known that the safety and reliability of pipeline transportation are crucial. We are aiming at the problem that the residual life and residual strength of the defective elbow pipes are difficult to predict and usually need to be obtained through experiments. Consequently, a combined method of numerical simulation technology combined with a genetic algorithm to optimize neural network extreme learning machine (GA-ELM) is proposed. Firstly, the erosion characteristics of elbow pipes with different defects under the conditions of different impurity particle flow rates, particle sizes, and mass flow rates are analyzed by numerical simulation. At the same time, the effects of erosion defects of different sizes on the equivalent stress and residual strength of elbow pipes are also studied. Based on numerical simulation data, the extreme learning machine prediction model optimized by a genetic algorithm is used to predict the erosion rate, residual life, and residual strength and compared with the traditional ELM network model. The results show that residual strength of the elbow pipes with the increase of the depth and length of the defect, and increases with the increase of the width of the defect; the GA-ELM model can not only effectively predict the erosion rate, residual life and residual strength of defective elbow pipes, moreover its prediction accuracy is better than the traditional ELM model.

## 1. Introduction

With the development of the economy and technology, our country’s demand for oil and gas resources is increasing. In the long-distance transportation of oil and gas products, pipeline transportation has a larger transportation volume, less land occupation, shorter construction period, lower cost, less energy consumption, and higher efficiency. Therefore, pipeline transportation is favored by people [1]. When oil and gas are transported at high speed and high pressure, centrifugal force will cause uneven erosion on the inner wall of the pipelines [2]. Consequently, with the increase in the service life of oil and gas pipelines, the reliability of pipelines will gradually decrease. In order to ensure the safety and reliability of oil and gas pipelines, it is necessary to study the residual life and residual strength of the pipelines and take reasonable measures to prevent the pipelines from being damaged.

In recent years, pipeline erosion fatigue has become a hot topic in domestic and foreign research [3,4,5,6,7,8,9]. Carlos used the finite element simulation method to study the influence of magazine particle concentration on elbow erosion and verified the accuracy of the model through experimental data [10]. In the study of Hong Bingyuan et al., a novel experimental device was designed to investigate the erosion characteristics of 304 stainless and L245 carbon steel in the gas-solid two-phase flow. Test results showed that the erosion can easily lead to wall thinning, perforation leakage, and other problems and cause significant safety risks to the safe operation of pipelines [11]. Zhang Yun developed the least-squares boosting model to predict the solid particle erosion rate in elbows from material properties, the geometry of the pipe wall and sand particles, as well carrier fluid velocities [12]. Ou Guofu used ANSYS software to establish a finite element model to study the vulnerable parts of the pipelines that are prone to damage under erosion [13]. Yang Yanhua used ABAQUS software to study the residual strength of oil and gas pipelines and wrote related programs to predict the failure probability of pipelines [14]. Zaidi et al. studied the structural integrity of welded pipes for oil drilling rigs and estimated the residual life of the pipes on this basis [15]. Hashim Abbas et al. established a neural network model to predict CO_2_ corrosion at high partial pressure [16]. Khalaj et al. presented some results of the research connected with the development of a new approach based on the artificial neural network (ANN) of predicting the ultimate tensile strength of the API X70 steels after thermomechanical treatment [17]. In the study of Khalaj et al., bainite fraction results of continuous cooling of high-strength low alloy steels had been modeled by artificial neural networks [18].

Most of the existing studies have carried out numerical simulation analysis or experimental research on defect-free pipelines to analyze their reliability and safety. The erosion effect of the particles mixed in the oil and gas on the pipe wall causes the residual life and residual strength of the pipelines to decrease rapidly, which affects the reliability of the entire pipeline transportation system. It is, however, important that few studies have proposed to study the remaining life and residual strength of oil and gas pipelines with defects. In order to predict and study the residual strength and residual life of defective oil and gas pipelines from the perspective of economy and safety, it is necessary to establish a scientific evaluation method. In this paper, the pipelines with erosion defects are taken as the research object. Combined with the engineering practice, the elbows with different erosion defects are simulated and compared. Erosion characteristics under different boundary conditions and the influence of different defect lengths, widths, and depths on the residual strength of erosion defect elbow pipes were analyzed. The prediction model of residual life and residual strength is verified by the numerical calculation results of the software and the experimental data in the literature, which provides an engineering reference for the prediction of the safety life of oil and gas pipelines with defects in practical applications.

## 2. Erosion Rate Analysis of 90° Elbow

### 2.1. Predictive Model for Erosion Wear

Oka of Hiroshima University proposed a new particle erosion model [19]. Compared with other existing erosion models, the Oka model takes more factors into accounts, such as relative velocity, particle diameter, and Vickers number of pipe materials, which will affect erosion wear.

(1) $$E(\theta )=g(\theta )E_{90}$$

(2) $$g(\theta )={\left(\sin\theta \right)}^{n_{1}}{\left[1+H_{v}(1-\sin\theta )\right]}^{n_{2}}$$

(3) $$E_{90}=K{\left(H_{v}\right)}^{k_{1}}{\left(\frac{u_{p}}{u_{pref}}\right)}^{k_{2}}{\left(\frac{d_{p}}{d_{pref}}\right)}^{k_{3}}$$

In Equations (1)–(3): E(θ) is the erosion rate, ${\mathrm{kg}/(m}^{2}\cdot s)$; g(θ) is the function of impact angle; $E_{90}$ is the reference erosion rate, ${\mathrm{kg}/(m}^{2}\cdot s)$; $H_{v}$ is the Vickers hardness of the eroded material, GPa; $u_{p}$ is the relative velocity between particle and wall, m/s; $u_{pref}=104\;m/s$ is the particle reference velocity constant; $d_{p}$ is the particle size of erosion particles, μm; $d_{pref}=326\;\mu m$ is the reference particle size; $n_{1}=0.8$, $n_{2}=1.3$ are exponents determined by the material hardness and other impact conditions such as particle properties; $k_{1}=-0.12$, $k_{2}=2.3{\left(H_{v}\right)}^{0.038}$, $k_{3}=0.19$ are exponent factors, which are affected by other parameters, respectively; K=65 denotes a particle property factor such as particle shape (angularity) and particle hardness, which has no correlation among different types of particles and other factors. Oka investigated the influence of impact parameters on the correlation equations of several aluminum, copper, carbon steel, and stainless steel specimens in detail and proved that the model could be used to estimate the experimental erosion damage data of various types of materials under various impact conditions [20]. This paper conducts calculations and analysis based on this model.

### 2.2. Model Parameters and Boundary Conditions

**Figure 1:**
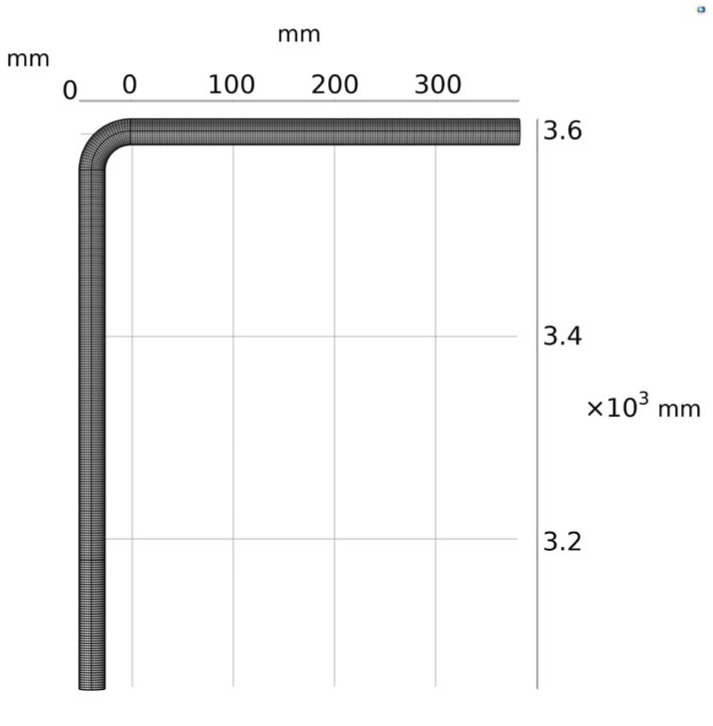
Elbow calculation model and mesh division.

Taking the experimental model in the literature [21] as an example, the elbow model, as shown in Figure 1, is established. The diameter of the pipe is 25.4 mm, the wall thickness is 12 mm, and the bend-diameter ratio is 1.5. Inside the pipeline is oil and gas with a gas density of 1.225 kg/m^3^ and a gas viscosity of 1.79 × 10^−5^ Pa·s, which flows in at the inlet at a speed of 34.1 m/s and flows out freely at the outlet. X100 pipeline steel is used as the material for the pipes. The particle size is 182 µm, and the particle-to-gas phase mass flow rate ratio is 0.013.

### 2.3. Mesh Division and Mesh Independence Verification

In CFD simulation, the calculation speed and accuracy are affected by the quality and quantity of the mesh. In this paper, considering both the calculation memory and the calculation accuracy, the free hexahedral mesh with the maximum element of 2.41 mm, the minimum element of 0.157 mm, and the maximum element growth rate of 1.08 is used for division. The total number of meshes divided is 134,928, as shown in Figure 1.

**Figure 2:**
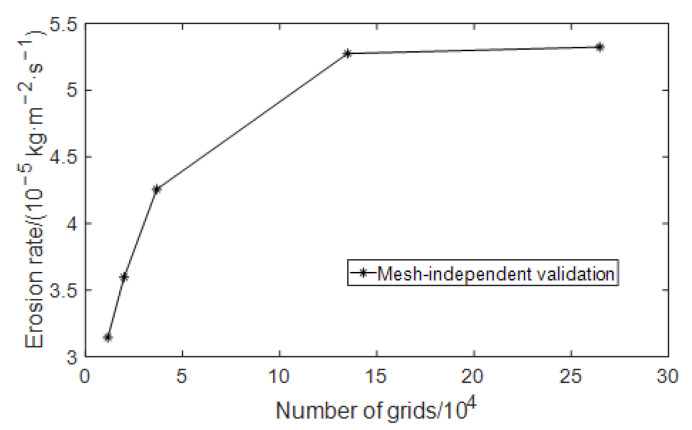
Mesh-independent validation of erosion rate model.

In order to verify the rationality of the mesh division mentioned above, the mesh independence is verified before the numerical simulation. When the mesh density reaches a certain degree, the calculation results tend to be stable. Increasing the number of mesh cells has little impact on the calculation results and increases the calculation amount and time. In this event, it can be considered that mesh independence has been achieved. In this paper, according to the mesh size gradient, models with mesh numbers of 11,598, 19,904, 36,715, 134,928, and 264,668 are selected for numerical simulation. The curve of the maximum erosion rate changing with the number of meshes is shown in Figure 2. It can be seen from Figure 2 that when the number of meshes reaches 134,928, the mesh independence requirement is met. Considering the amount of calculation and calculation accuracy at the same time, the number of meshes for subsequent simulations is at least 130,000.

### 2.4. Validation of Numerical Methods

**Figure 3:**
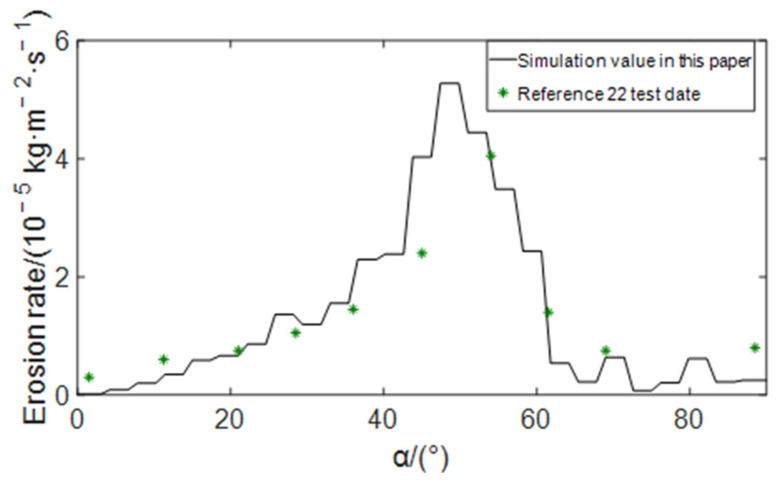
Comparison of erosion rate distribution at the elbow.

The comparison between the simulation results of this paper and the experimental data of Mazumder et al. [22] is shown in Figure 3. It can be seen from the figure that the distribution curve of erosion rate at the elbow obtained by simulation calculation in this paper can be well fitted with the test data, indicating that the mathematical model and simulation method adopted in this paper are reasonable and feasible.

**Figure 4:**
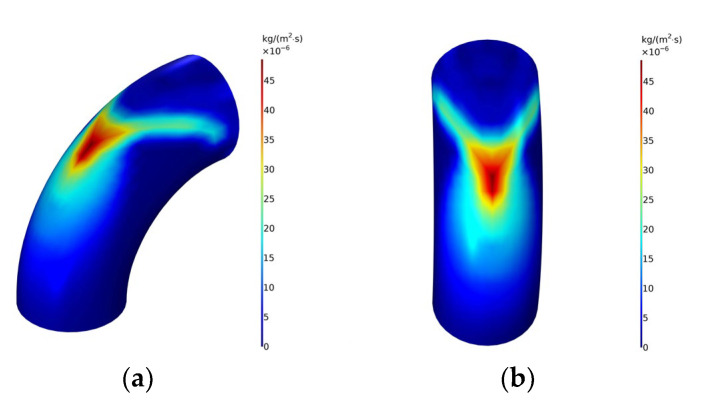
Schematic diagram of erosion rate distribution at elbows. (**a**) Side view; (**b**) Front view.

The schematic diagram of the pipeline erosion rate is shown in Figure 4. It can be seen from the figure that V-shaped erosion wear occurs at the outermost side of the elbow, with the highest erosion rate. There is a drag force in the fluid flow process. When flowing through the elbow, the solid particles doped in the fluid will impact the pipe wall under the action of inertia, resulting in the above phenomenon.

## 3. Erosion Rate Analysis of Defective Elbow Pipes

### 3.1. Model Parameters

**Figure 5:**
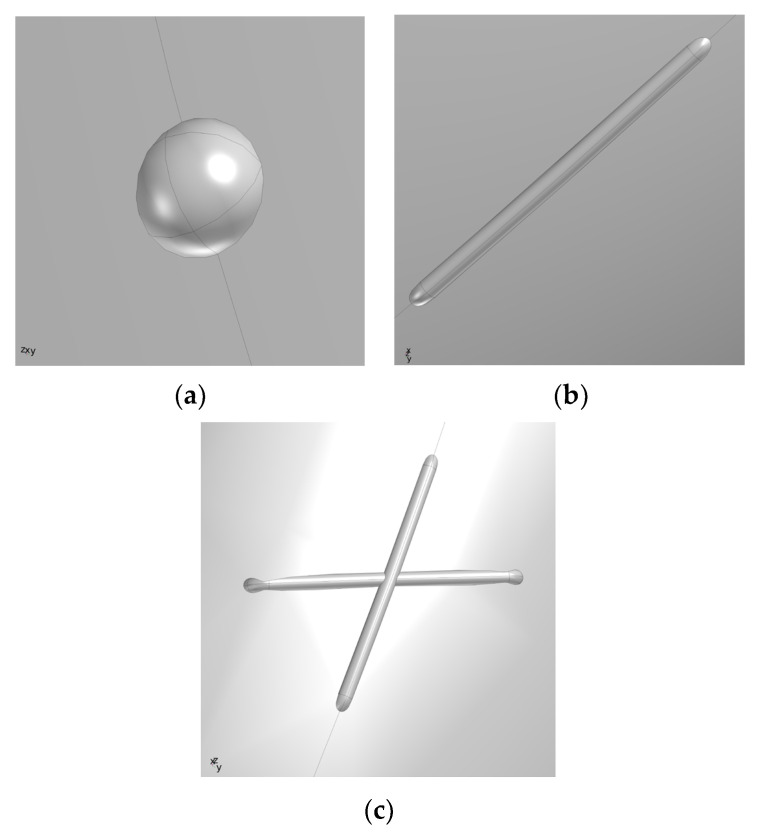
Defect location and partial local enlarged map of the elbow. (**a**) Schematic diagram of point defect; (**b**) Schematic diagram of trench defect; (**c**) Schematic diagram of double trench defect.

On the basis of the model in Figure 4, combined with the defect shape and wall thickness loss in literature [21], the point defect, groove defect, and double groove defect with a depth of 200 µm were drawn at the elbow with the most severe erosion wear. As shown in Figure 5.

### 3.2. Analysis of Simulation Results

**Figure 6:**
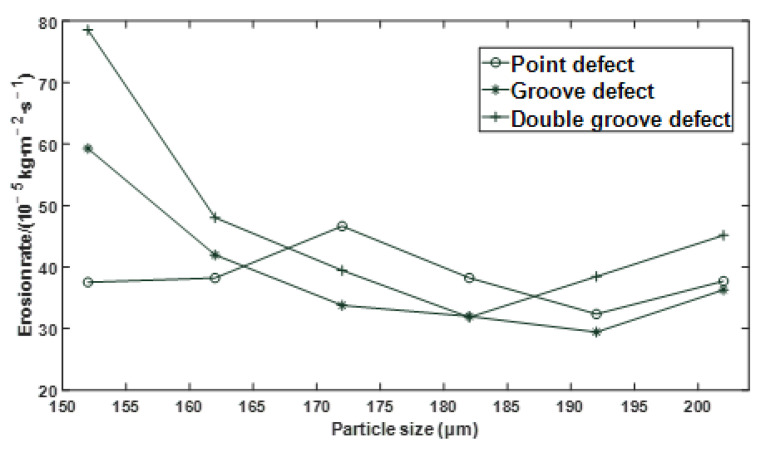
Effect of particle size on erosion rate under different defect conditions.

**Figure 7:**
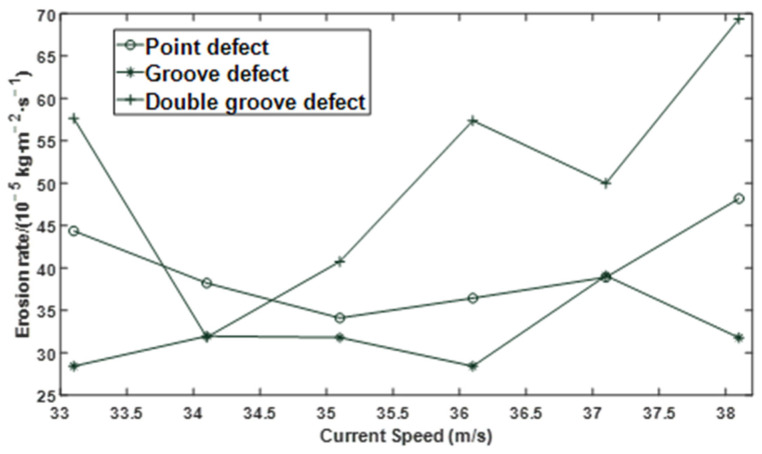
Effect of flow velocity on erosion rate under different defect conditions.

**Figure 8:**
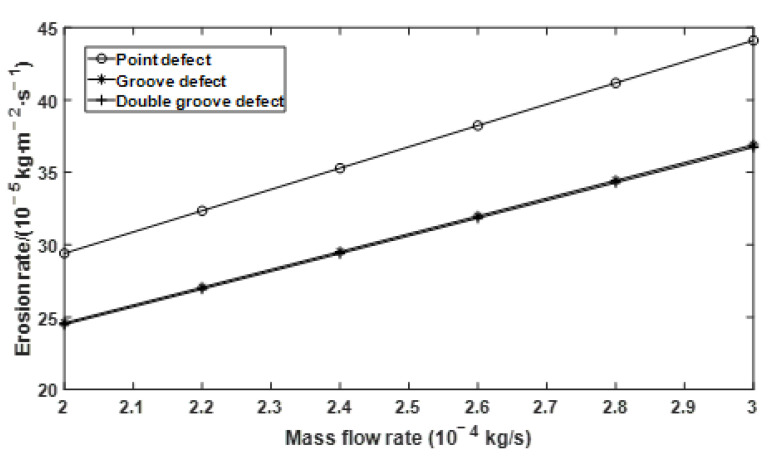
Effect of mass flow rate on erosion rate under different defect conditions.

The effects of particle size, flow velocity, and mass flow rate on the erosion rate of the defective elbows were studied, respectively. When the particle size is a variable, the flow velocity is 34.1 m/s, and the particle-to-gas mass flow rate ratio is 0.013; When the flow velocity is a variable, the particle size is 182 µm, and the particle and gas phase mass flow rate is 0.013; When the ratio of particle to gas phase mass flow rate is a variable, the flow velocity is 34.1 m/s, and the particle size is 182 µm. The changing trend of erosion rate of point defect, groove defect, and double groove defect under the boundary conditions of different particle size, flow velocity, and mass flow rate is shown in Figure 6, Figure 7 and Figure 8.

It can be seen from Figure 6 and Figure 7 that under different defect conditions, the particle size and flow velocity are nonlinear with the erosion rate.

It is known from Figure 8 that under three different defect conditions, the erosion rate increases with the increase of mass flow rate. Erosion rate curves of groove defect and double groove defect almost overlap, which is smaller than that of point defect in numerical value.

## 4. Analysis of Residual Strength of Defective Elbow Pipes

### 4.1. Model Parameters and Boundary Conditions

**Figure 9:**
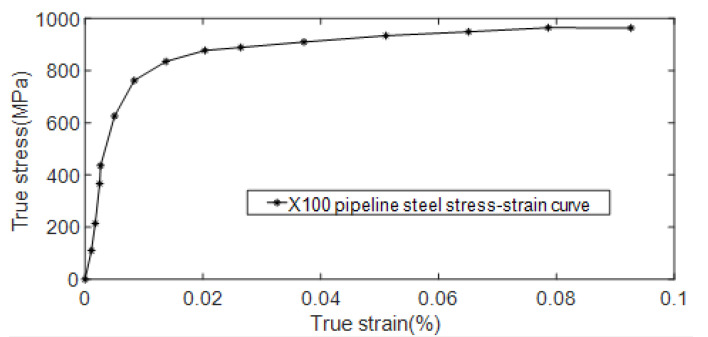
X100 pipeline steel true stress-strain curve.

Using X100 pipeline steel as the material, a three-dimensional elbow model with a pipe diameter of 1320 mm and a wall thickness of 22.9 mm was established. X100 pipeline steel is widely used in the field of oil and gas resources transportation with good performance and low cost. The hardening effect of pipeline steel has a great influence on the residual strength of high-strength pipelines. In the process of numerical simulation, it is necessary to explore the problem of pipeline nonlinearity. In order to accurately describe the change of pipe section area in the process of large deformation of pipes, when defining X100 pipe material properties, not only Poisson’s ratio, elastic modulus, and other linear parameters of pipes are set but also real stress and plastic strain are considered. The real stress-strain data of X100 pipeline steel are input in the material properties as known conditions when the model is established. The performance parameters of X100 pipeline steel to be set in this paper are shown in Table 1, the chemical composition is shown in Table 2, and the true stress-strain curve of X100 pipeline steel is shown in Figure 9.

**Figure 10:**
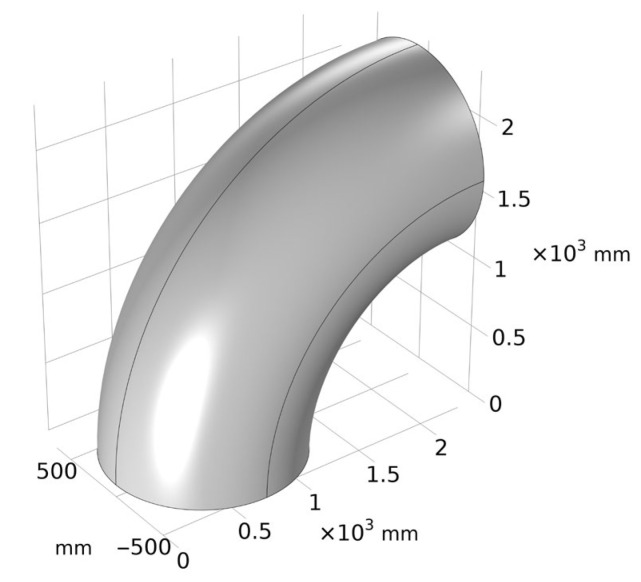
Calculation model of bending tube.

In order to simplify the actual erosion model, certain assumptions are made, and the applied loads and boundary conditions are set as follows:

(1) The coupling effect of pipe gas and pipe soil (such as the friction between pipe gas and pipe soil, the velocity of the medium in the pipe, etc.) during the operation of the pipelines is not considered; the thermal expansion and cold contraction due to changes in ambient temperature are not considered The thermal stress generated in the pipelines when the phenomenon is limited by the internal and external constraints of the pipelines; the force generated by the anti-erosion protection measures of the pipelines are not considered.
(2) Only the effect of the internal pressure on the pipelines during the operation of the pipelines is considered, and the direction of the force is perpendicular to the inner surface of the pipelines; the forces generated by the pipelines’ own weight, bending moment, and seismic load are not considered.
(3) Displacement constraints are imposed on the left and right ends of the model; that is, the ends are completely fixed. The elbow model is shown in Figure 10.

When analyzing volumetric defects, certain failure criteria need to be established. In this paper, the plastic failure criterion is used to judge whether the pipelines fail. When the Von Mises equivalent stress in the defect area exceeds the ultimate tensile strength, the pipeline is considered to be invalid. The internal pressure that the pipelines can bear at this time is the residual strength of the pipelines. The Von Mises equivalent stress calculation formula is as follows.

(4) $$\sigma_{\theta }=\sqrt{\frac{1}{2}\left[{\left(\sigma_{1}-\sigma_{2}\right)}^{2}+{\left(\sigma_{2}-\sigma_{3}\right)}^{2}+{\left(\sigma_{3}-\sigma_{1}\right)}^{2}\right]}$$

Among them, $\sigma_{\theta }$ represents the Von Mises equivalent stress (MPa); $\sigma_{1}$, $\sigma_{2}$ and $\sigma_{3}$ represent the stress (MPa) along the X-axis, the Y-axis, and the Z-axis direction, respectively [23,24,25].

### 4.2. Meshing and Mesh Independence Verification

**Figure 11:**
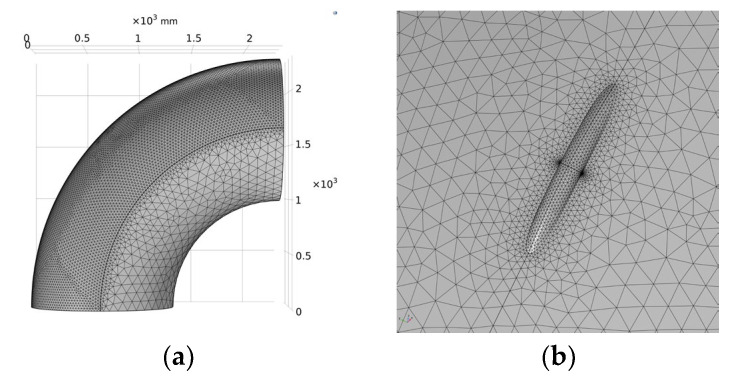
Mesh Schematic. (**a**) Overall meshing; (**b**) Meshing at defects.

In this paper, the free tetrahedron mesh with a maximum element of 80.8 mm, a minimum element of 3.46 mm, and a maximum element growth rate of 1.35 is used to divide the elbow model. The total number of meshes divided is 85,258, as shown in Figure 11.

**Figure 12:**
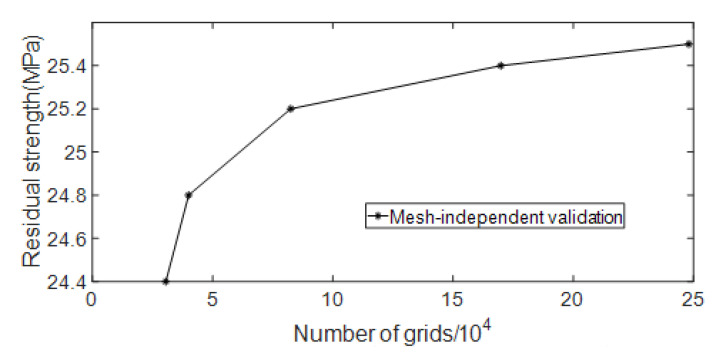
Mesh-independent validation of strength model.

According to the mesh size gradient, models with mesh numbers of 30,521, 40,018, 85,258, 169,919 and 248,087 are selected for numerical simulation. The curve of residual strength changing with the number of meshes is shown in Figure 12. It can be seen from the figure that mesh independence is achieved when the number of meshes reaches 85,258. Considering the amount of calculation and calculation accuracy at the same time, the number of meshes for subsequent simulations is at least 80,000.

### 4.3. Numerical Method Validation

Collect the real data of seven groups of pipeline blasting tests with defects in the literature [26,27,28] and calculate the residual strength of pipelines with defects with different parameters according to the established finite element model. The specific parameters and calculation results are shown in Table 3.

By comparing the residual strength predicted by the finite element model with the blasting data provided in the literature, all the predicted residual strength values are within ±10% of the deviation of the actual residual strength, the minimum relative error is 0.3%, and the maximum relative error is 9.5%. It shows that the established nonlinear finite element model can more accurately predict the residual strength of oil and gas pipelines with defects, and at the same time, verifies that the established nonlinear finite element model, mesh division, and selected failure criteria are reasonable and feasible.

### 4.4. Analysis of Simulation Results

**Figure 13:**
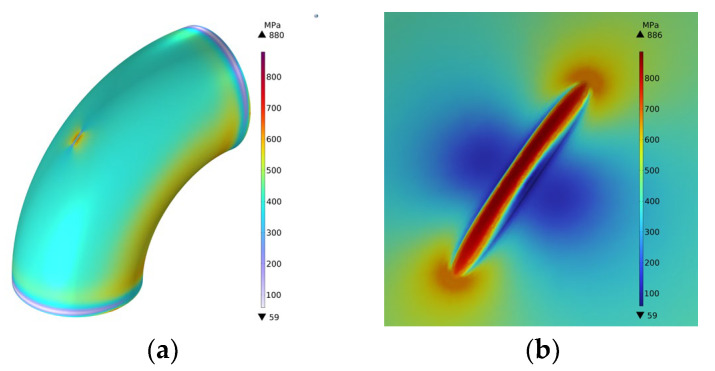
Stress diagram. (**a**) Overall stress diagram; (**b**) Schematic diagram of stress at the defect.

**Figure 14:**
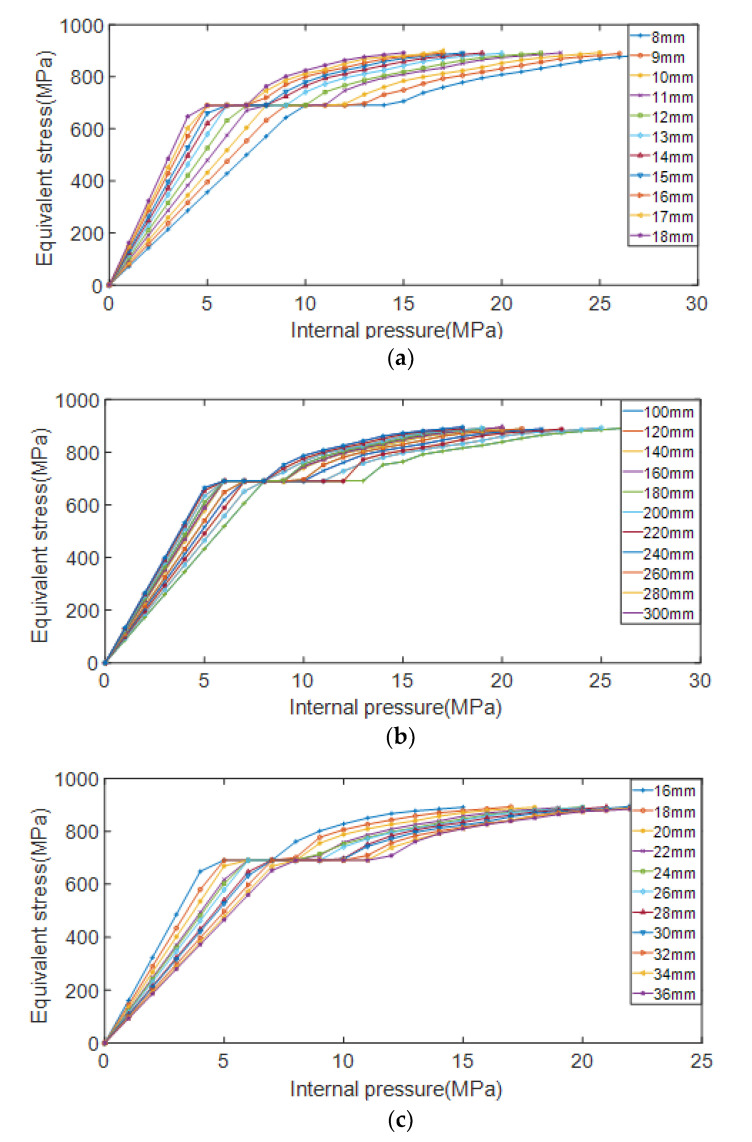
Variation of equivalent stress with internal pressure load under different defect conditions. (**a**) Different defect depths; (**b**) Different defect lengths; (**c**) Different defect widths.

Taking the pipelines with groove-shaped erosion defects as the research object, the influence of different geometric parameters on the residual strength of the pipelines was studied. Taking the defect length *l* = 200 mm, the defect width *w* = 26 mm, and the defect depth *d* = 13 mm as an example, when the equivalent stress in the defect area reached the ultimate tensile strength of 886 MPa, the schematic diagram of the equivalent stress was as shown in Figure 13:

The pipelines with different depths, lengths, and widths of erosion defects were simulated and analyzed, respectively. When the defect depth *d* is taken as a variable, the length of the control defect is *l* = 200 mm, the width *w* = 26 mm is unchanged, and the depth of the defect is *d* = 8, 9, 10, 11, 12, 13, 14, 15, 16, 17, 18 mm. When the defect length *l* is taken as a variable, the control defect depth *d* = 13 mm, the width *w* = 26 mm remains unchanged, and the defect length is *l* = 100, 120, 140, 160, 180, 200, 220, 240, 260, 280, 300 mm in turn. When the defect width *w* is taken as a variable, the control defect depth *l* = 200 mm, the depth *d* = 13 mm remains unchanged, and the defect width is sequentially taken as *w* = 16, 18, 20, 22, 24, 26, 28, 30, 32, 34, 36 mm. The variation curve of von Mises equivalent stress with the increase of internal pressure load under different erosion defect length, width and depth conditions is shown in Figure 14.

It can be seen from Figure 14 that the equivalent stress of the pipelines obviously has two stages with the change of the internal pressure, namely the elastic stage of the pipe and the plastic expansion stage of the pipe. In the elastic deformation stage, the larger the defect depth *d* and the larger the defect length *l*, the faster the equivalent stress of the pipelines increases with the internal pressure; the larger the defect width *w*, the slower the equivalent stress of the pipelines increases with the internal pressure. In the strengthening stage after the pipelines have yielded, the curves in the figure are almost parallel, and the equivalent stress under the condition of different geometric parameters of the defect tends to be equal with the growth rate of the internal pressure load. So the plastic strengthening effect of the pipeline steel will not decrease with the change of the geometric parameters, delaying the effect of defects on residual strength.

**Figure 15:**
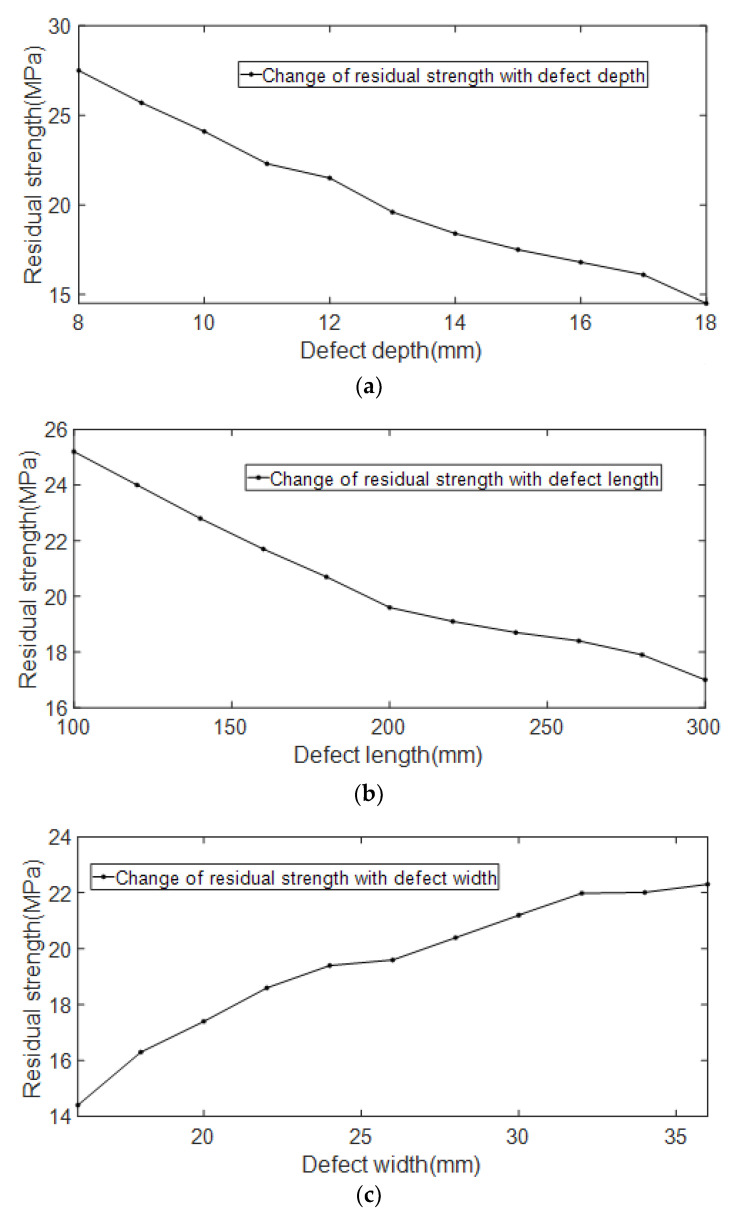
Relationship between defect geometry parameters and residual strength. (**a**) Schematic diagram of the relationship between residual strength and defect depth; (**b**) Schematic diagram of the relationship between residual strength and defect length; (**c**) Schematic diagram of the relationship between residual strength and defect width.

The relationship between the residual strength of the pipelines and the length, width, and depth of the erosion defect is shown in Figure 15.

It can be seen from Figure 15 that with the increase of the depth and length of the erosion defect, the residual strength shows an obvious linear decreasing trend. As the width of the erosion defect increases, the residual strength shows an obvious linear increasing trend. In terms of numerical changes, the change in defect width has less effect on the residual strength of the pipelines than the depth and length of the defect.

## 5. Prediction of Erosion Life and Residual Strength

### 5.1. Extreme Learning Machine

**Figure 16:**
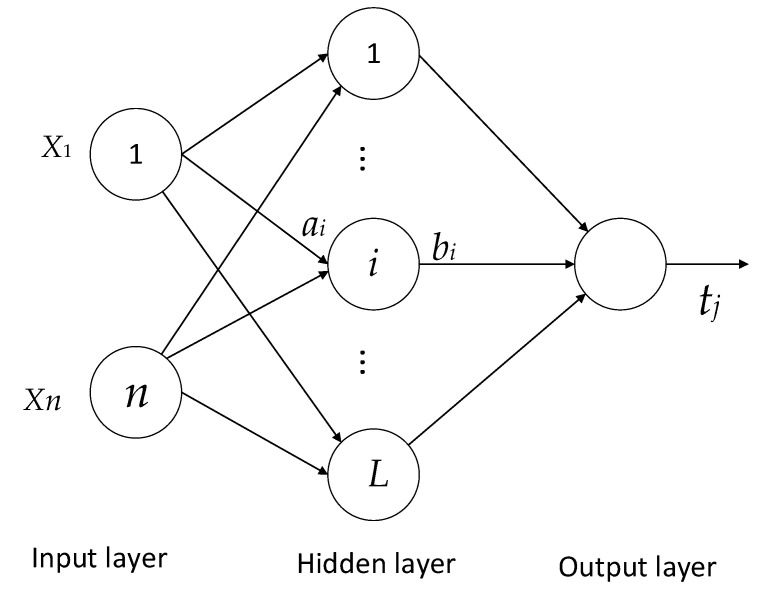
ELM network structure.

Extreme Learning Machine (ELM) is a new type of single-hidden layer feedforward neural network algorithm. Its hidden layer node parameters are randomly selected, the external network weight obtains its least squares solution by minimizing the squared loss function, and the process of determining network parameters does not require any iterative steps, which improves the calculation speed. ELM is simple and easy to implement, which overcomes the shortcomings of the traditional neural network based on the gradient descent method that the training speed is slow and easily falls into local optimum. The network structure of ELM is shown in Figure 16, the number of input layers is *n*, and the number of hidden layers is *L*. Its working principle is as follows:

Given the training sample set ${\left\{\left(x_{i},t_{i}\right)\right\}}_{i=1}^{N}$ and the number of hidden layer neurons *L*, the presence of $a_{i}$, $b_{i}$, $\beta_{i}$ makes:

(5) $$f_{L}\left(x_{j}\right)=\sum_{i=1}^{L}\beta_{i}g\left(a_{i}x_{j}+b_{i}\right)=t_{j}$$

Among them: $a_{i}$ and $b_{i}$ are hidden layer node parameters; $\beta_{i}$ represents the outer weight between the *i*-th hidden layer node and the network output; *g* is the activation function.

Equation (5) is written in matrix form as follows:

(6) Hβ=T

The specific details are as follows: $H=H\left(a_{1},\cdots ,a_{L},b_{1},\cdots ,b_{L},x_{1},\cdots ,x_{N}\right)={\left[\begin{array}{c} g\left(a_{1}x_{1}+b_{1}\right)\\ \vdots \\ g\left(a_{1}x_{N}+b_{1}\right) \end{array}\begin{array}{c} g\left(a_{2}x_{1}+b_{1}\right)\\ \vdots \\ g\left(a_{2}x_{N}+b_{1}\right) \end{array}\begin{array}{c} \cdots \\ \cdots \end{array}\begin{array}{c} g\left(a_{L}x_{1}+b_{L}\right)\\ \vdots \\ g\left(a_{L}x_{N}+b_{L}\right) \end{array}\right]}_{N\times L}$, $\beta ={\left[\begin{array}{c} \beta_{1}^{T}\\ \vdots \\ \beta_{L}^{T} \end{array}\right]}_{L\times m}$, $T={\left[\begin{array}{c} t_{1}^{T}\\ \vdots \\ t_{N}^{T} \end{array}\right]}_{N\times m}$.

*H* is the hidden layer output matrix. Considering the prediction error, equation (6) can be modified to:

(7) Hβ=T+E

Defining the squared loss function:

(8) $$J={\left(H\beta -T\right)}^{T}\left(H\beta -T\right)$$

Then the training problem of the ELM network parameters is transformed into a minimized squared loss function problem; that is, it is necessary to find a set of optimal parameters ${\left\{\left(a_{i},b_{i},\beta_{i}\right)\right\}}_{i=1}^{L}$ such that *J* is minimal. When the function *g* is activated infinitely differentiable, the hidden layer node parameters can be randomly selected at the beginning of training, fixed during training, and the external weight β can be obtained by solving the system of Equation (8) by the least squares method:

(9) $$\overset{\wedge }{\beta }=\arg\min\left\|H\beta -T\right\|=H^{+}T$$

ELM only needs to select the number of hidden layer nodes under the condition of determining the activation function, and the parameter determination process is relatively simple. The specific solution steps are as follows:

(1) Set the hidden layer activation function $g\left(x\right)$ and the number of hidden layer nodes *L*;
(2) Randomly generate hidden layer node parameters $\left(a,b_{i}\right)$, i=1,2,⋯L ;
(3) Calculate the hidden layer output matrix *H*;
(4) Calculate the output weight $\overset{\wedge }{\beta }=H^{+}T$, where $H^{+}$ is the generalized inverse of Moore–Penrose of the matrix *H*.

### 5.2. Genetic Algorithm

Genetic Algorithm (GA) is an adaptive probability search method formed by simulating the genetic mechanism and biological evolution process in nature, with inherent hidden parallelism and global optimization ability. It maps the solution problem to the bit string space, represents a potential solution set of the problem as a population, and the solution of the problem as a chromosome, that is, an individual in the population, and performs survival of the fittest based on the fitness function. Genetic algorithms evolve populations through a series of operators, producing new offspring. Standard genetic operators include selection operators, cross operators, and mutation operators. The genetic algorithm operation process consists of the following steps:

(1) Encoding.

The solution data of the solution space is represented as the genotype string structure data of the genetic space, and different combinations of string data represent different solutions to the problem. The commonly used coding methods are Binary coding, Gray code coding, and One-hot coding.

(2) Population initialization.

Determine population size N, cross probability $P_{c}$, mutation probability $P_{m}$, and termination evolution criterion, randomly generate N individuals as the initial population $X\left(0\right)$, set the current evolutionary algebra k=0, and the maximum evolutionary algebra is Y.

(3) Calculate the fitness value.

The fitness value indicates the merits of an individual or solution, and different fitness functions are defined for different problems, and the fitness value of ${\left\{x_{k}^{i}\right\}}_{i=1}^{N}$ is calculated for each individual in the k-generation population.

(4) Genetic manipulation.

Select, cross, and mutant operators act on the current population in turn to achieve evolution.

(5) Check the termination conditions.

If the genetic algebra satisfies the termination condition, the calculation is terminated, and the best individual in the current population is taken as the final satisfactory solution output; Otherwise, k=k+1 and go to step (3).

**Figure 17:**
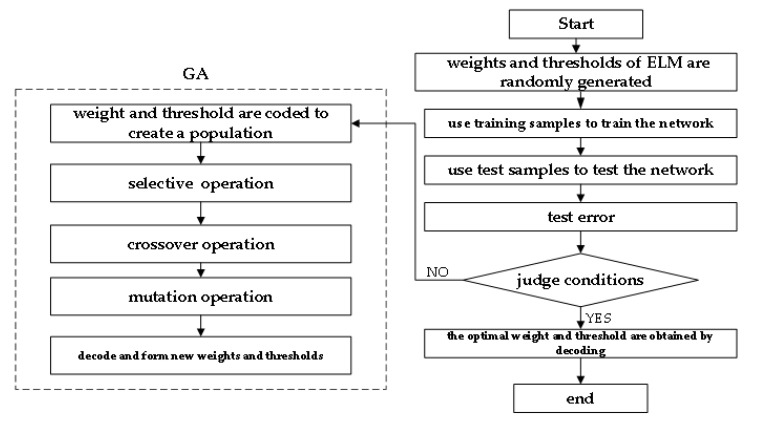
Workflow chart of GA-ELM mode.

The workflow chart of the extreme learning machine optimized by genetic algorithm (GA-ELM) is shown in Figure 17.

### 5.3. Prediction Results and Analysis of Test Data

In this paper, MATLAB software is used to program and simulate the GA-ELM model, and 61 groups of blasting test data of defective pipelines in the literature [29] are selected to verify the accuracy of the model. Among them, 50 groups were randomly selected as training sets, and 11 groups were used as prediction sets. Some data are shown in Table 4.

The prediction results of the test set are shown in Table 5. It can be seen from the table that the minimum relative error of GA-ELM model prediction results is 1.75%, the maximum relative error is 13.78%, and the average relative error is 5.05%.

**Figure 18:**
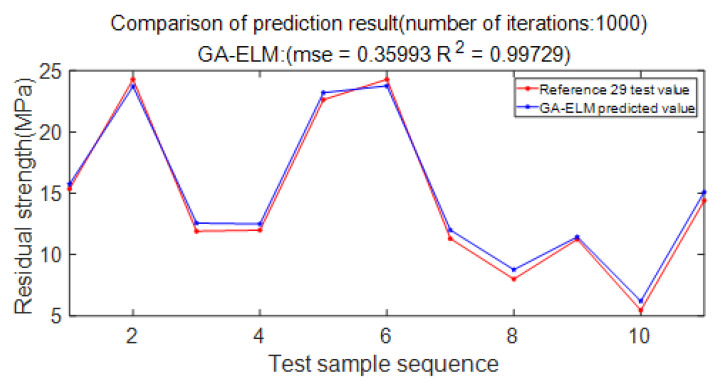
Comparison of forecast results.

Figure 18 shows the comparison between the test values in the literature and the predicted values of the GA-ELM model. It is known from the figure that after 1000 iterations, the prediction accuracy of the GA-ELM model reaches 99.729%, which can better fit the nonlinear relationship between the residual strength of the defective pipelines and its influencing factors. It is proved that the prediction model adopted in this paper is reasonable and feasible.

### 5.4. Prediction Results and Analysis of Simulation Data

According to the erosion rate obtained by numerical simulation, the residual life of the pipelines can be calculated by Equation (10) [30]. In the Equation: h is the safe allowable wall thickness of the pipe wall, mm; ρ is the density of X100 pipeline steel, ${\mathrm{kg}/m}^{3}$; R is the erosion rate, ${\mathrm{kg}/(m}^{2}\cdot s)$.

(10) $$T=\frac{h\rho }{R}$$

In this paper, 300 sets of erosion rate and erosion life data of elbows without defects, point defects, and groove defects are obtained through simulation calculation, and each set of data contains 5 types. No defect is set as working condition 1, point defect is set as working condition 2, and groove defect is set as working condition 3. With the working condition, particle size, flow velocity, and mass flow rate as the input, and the erosion rate or residual life as the output. By training the neural network, the prediction of the erosion rate and the residual life of the defective elbow can be realized. In total, 250 sets of data were randomly selected as the training set, and the remaining 50 sets of data were used as the test set. Part of the sample data is shown in Table 6.

In this paper, the residual strength data of 93 sets of pipelines with erosion defects obtained by simulation are used, and each set of data contains eight types. The pipe diameter, wall thickness, yield strength, tensile strength, notch length, notch width, and notch depth are used as input, and the residual strength is used as output. In total, 73 sets of data were randomly selected as the training set, and the remaining 20 sets of data were used as the test set. Part of the sample data is shown in Table 7.

**Figure 19:**
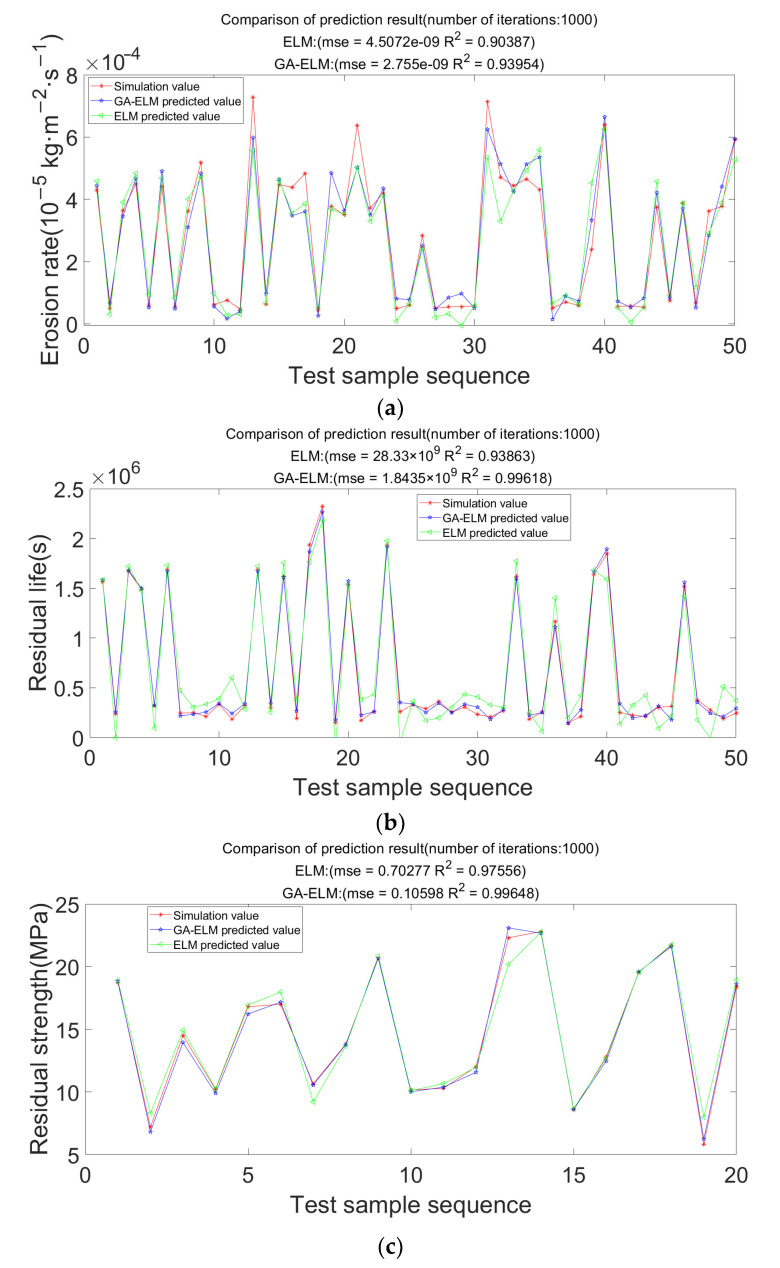
Comparison of the accuracy of the two prediction models. (**a**) Schematic diagram of erosion rate prediction; (**b**) Schematic diagram of residual life prediction; (**c**) Schematic diagram of residual strength prediction.

The comparison between the simulation value and the prediction results of the two models is shown in Figure 19. It is known from the figure that the prediction accuracy of the ELM model for the erosion rate, residual life, and residual strength is 90.387%, 93.863%, and 97.556%. The accuracy of the GA-ELM model is 93.954%, 99.618%, and 99.648%, which are higher than the ELM model. It proved that this optimization algorithm could significantly improve the prediction accuracy.

## 6. Conclusions

In this paper, taking the defective elbow pipes as the research object, the numerical simulation method is used to analyze the influence of the flow rate, particle size, and mass flow rate of solid particles on the erosion characteristics of the elbow with defects. In addition, this paper studies the influence of defect length, width, and depth on residual strength. Furthermore, it combines artificial intelligence algorithms to predict the residual life and residual strength of erosion. The above calculation results show that:

(1) As has been noted, the simulation results are in good agreement with the published experimental data. The average errors of maximum erosion rate and residual strength prediction are 12.7% and 3.7%, respectively, which are within the acceptable range. Therefore, it is verified that the mathematical model adopted in this paper can effectively calculate the erosion rate and residual strength of the defective elbow pipes.

(2) In this study, the maximum erosion rate of the elbow with defects has a positive linear relationship with the mass flow rate of solid particles. In contrast, it has a nonlinear relationship with flow rate and particle size. The maximum equivalent stress of the defective elbow pipes can be divided into the yield stage and the plastic strengthening stage. The residual strength of the defective elbow pipes is negatively correlated with the depth and length of the defect, whereas positively correlated with the width of the defect relation.

(3) As described above, the extreme learning machine GA-ELM model optimized by the genetic algorithm in this paper is reasonable and feasible, and the prediction accuracy of the blasting test data of pipelines with defects reached 99.729%. The accuracy of the GA-ELM model in predicting the erosion rate, residual life, and residual strength of the elbow with defects is 93.954%, 99.618% and 99.648%, respectively. Compared with the traditional extreme learning machine ELM model, the prediction accuracy is effectively improved by 3.567%, 5.755%, and 2.092%, respectively.

## Figures and Tables

**Table 1:** Performance parameters of X100 pipeline steel.

Minimum Yield Strength (MPa)	Minimum Tensile Strength (MPa)	Ultimate Tensile Strength (MPa)	Young’s Modulus (MPa)	Poisson’s Ratio
690	760	886	210,000	0.3

**Table 2:** Chemical composition of X100 pipeline steel (%).

C	Si	Mn	P	S	Cr	Ni	Mo	Fe
0.065	0.95	1.69	0.015	0.002	0.04	0.03	0.27	allowance

**Table 3:** Prediction and comparison of residual strength of pipeline steel under different notch conditions.

Number	Pipe Diameter (mm)	Wall Thickness (mm)	Defect Length (mm)	Defect Depth (mm)	Residual Strength (MPa) (Literature Test Value)	Residual Strength (MPa) (Simulation Results)	Error (%)
1	1320	22.9	200	4.58	27.79	27.87	0.3
2	1320	22.9	1000	11.45	16.59	16.64	0.3
3	1320	22.9	514.98	11.45	17.9	17.72	1.0
4	1320	22.9	1109.93	11.45	15.4	16.01	4.0
5	1320	22.9	556.88	11.36	18.1	17.3	4.4
6	1320	22.9	1012.74	11.45	15	15.97	6.4
7	1320	22.9	800	41.2	18.76	16.97	9.5

**Table 4:** Blasting test data of pipelines with defects.

Pipe Diameter (mm)	Wall Thickness (mm)	Defect Length (mm)	Defect Depth (mm)	Defect Width (mm)	Yield Strength (MPa)	Tensile Strength (MPa)	Residual Strength (MPa)
458.8	8.1	39.6	5.39	31.9	601	684	22.68
459.4	8	40.05	3.75	32	589	730.5	24.2
323.9	9.8	255.6	7.08	95.3	452	542	14.4
323.9	9.66	305.6	6.76	95.3	452	542	14.07
508	14.6	500	10.35	97	478	600	14.6
508	14.3	500	10.3	97	478	600	13.4
76.2	2	75	1.4	16	391	458	9.4
76.2	2.04	75	1.44	16	260	309	5.45
762	17.5	200	9	200	474.1	556.6	22.64
426	6.95	160	2.7	25	240	390	10.8
…	…	…	…	…	…	…	…
426	7	150	3.8	21	240	390	9.81
529	9	350	4.7	25	285	415	8.83
529	9	160	4.7	25	285	415	15.7
720	8	320	4.4	26	425	535	8.83
720	8	180	6.2	26	425	535	7.55
304.8	6.35	26	4.95	20	351	543	15.36
304.8	6.35	33	4.25	21	382	570	16.29
323.9	9.74	527.8	7.06	95.3	422.5	589.6	11.3
1422.4	19.25	180	10.4	0.5	740	774	15.35
914.4	16.4	450	6	0.5	739	813	24.02
…	…	…	…	…	…	…	…
304.8	6.35	37	4.64	30	351	463	14.29
324	6.01	19.35	3.6	19	382	570	16.22
324	10.3	243	5.15	154.5	380	514	23.2
324	10.3	243	5.15	30.9	380	514	22
508	6.6	381	2.62	35.4	443.4	598.9	11.25
508	6.35	900	3.43	25.4	429.6	572.5	8
323.9	9.79	500	6.99	95.3	452	542	11.99
323.9	9.74	527.8	7.14	95.3	452	542	11.3
762	17.5	200	8.4	100	474.1	556.6	23.42
762	17.5	200	9	200	474.1	556.6	22.64

**Table 5:** The test set prediction results.

Number	Test Values in Literature (MPa)	Predicted Value of GA-ELM (MPa)	Error (%)
1	15.36	15.7528	2.56
2	24.3	23.7243	2.37
3	11.91	12.5701	5.55
4	11.99	12.4956	4.22
5	22.64	23.2093	2.51
6	24.3	23.7668	2.19
7	11.3	12.0015	6.21
8	8	8.7686	9.61
9	11.25	11.4471	1.75
10	5.45	6.2013	13.78
11	14.4	15.0982	4.85

**Table 6:** Erosion rate and residual life sample data.

Defect	Velocity of Flow (m/s)	Mass Flow Rate (10^−4^ kg/s)	Grain Size (µm)	Maximum Erosion Rate (10^−5^ kg∙m^−2^∙s^−1^)	Residual Life Span (10^5^ s)
0	34.1	2.60	152	5.10	18.5
0	34.1	2.80	152	5.49	17.1
0	33.1	2.60	152	4.74	19.9
0	33.1	2.60	152	5.10	18.5
0	35.1	2.60	152	5.44	17.3
…	…	…	…	…	…
0	37.1	2.20	202	5.56	17.0
0	37.1	2.40	202	6.06	15.5
0	38.1	2.60	202	6.99	13.5
0	38.1	2.80	202	7.53	12.5
0	38.1	3.00	202	8.06	11.7
1	33.1	2.60	142	83.0	1.13
1	34.1	2.60	142	35.8	2.63
1	35.1	2.60	142	28.7	3.28
1	36.1	2.60	142	54.8	1.72
1	33.1	2.80	142	27.6	3.41
…	…	…	…	…	…
1	37.1	2.00	202	35.4	2.66
1	37.1	2.20	202	39.0	2.42
1	37.1	2.40	202	42.5	2.22
1	38.1	2.80	202	52.6	1.79
1	38.1	3.00	202	56.4	1.67
2	35.1	2.60	142	47.3	1.99
2	36.1	2.60	142	72.8	1.29
2	33.1	2.60	142	59.3	1.59
2	34.1	2.60	142	66.2	1.42
2	34.1	2.80	142	71.3	1.32
…	…	…	…	…	…
2	37.1	3.20	202	45.9	2.05
2	37.1	3.00	202	43.0	2.19
2	38.1	2.80	202	44.4	2.12
2	38.1	2.40	202	38.1	2.48
2	38.1	2.20	202	34.9	2.70

**Table 7:** Residual intensity sample data.

Pipe Diameter (mm)	Wall Thickness (mm)	Defect Length (mm)	Defect Depth (mm)	Defect Width (mm)	Yield Strength (MPa)	Tensile Strength (MPa)	Residual Strength (MPa)
1320	22.9	100	13	26	690	886	25.2
1320	22.9	120	13	26	690	886	24
1320	22.9	140	13	26	690	886	22.8
1320	22.9	160	13	26	690	886	21.7
1320	22.9	180	13	26	690	886	20.7
…	…	…	…	…	…	…	…
1320	22.9	200	14	26	690	886	18.4
1320	22.9	200	15	26	690	886	17.5
1320	22.9	200	16	26	690	886	16.8
1320	22.9	200	17	26	690	886	16.1
1320	22.9	200	18	26	690	886	14.5
1422.4	20.1	100	11	22	795	840	15.7
1422.4	20.1	120	11	22	795	840	14.67
1422.4	20.1	140	11	22	795	840	13.8
1422.4	20.1	160	11	22	795	840	12.78
1422.4	20.1	180	11	22	795	840	12.3
…	…	…	…	…	…	…	…
1422.4	20.1	200	12	22	795	840	11.2
1422.4	20.1	200	13	22	795	840	10.01
1422.4	20.1	200	14	22	795	840	9.31
1422.4	20.1	200	15	22	795	840	8.57
1422.4	20.1	200	16	22	795	840	8.2
914.4	16.4	100	10	20	739	813	15.4
914.4	16.4	120	10	20	739	813	14.1
914.4	16.4	140	10	20	739	813	13.21
914.4	16.4	160	10	20	739	813	12.5
914.4	16.4	180	10	20	739	813	12.2
…	…	…	…	…	…	…	…
914.4	16.4	200	11	20	739	813	10.2
914.4	16.4	200	12	20	739	813	9.4
914.4	16.4	200	13	20	739	813	8.7
914.4	16.4	200	14	20	739	813	7.2
914.4	16.4	200	15	20	739	813	5.83

## Data Availability

Data is available in the paper.

## Nomenclature

E(θ)	corrosion rate (kg/m^2^·s)
g(θ)	function of impact angle
$E_{90}$	reference wear rate (kg/m^2^·s)
$H_{v}$	Vickers hardness of the eroded material
$u_{p}$	relative velocity between particle and wall (m/s)
$d_{p}$	particle size of erosion particles (μm)
$u_{pref}$	particle reference velocity constant
$d_{pref}$	reference particle size
$\sigma_{\theta }$	Von Mises equivalent stress (MPa)
$\sigma_{1}$	the stress along the X-axis (MPa)
$\sigma_{2}$	the stress along the Y-axis (MPa)
$\sigma_{3}$	the stress along the Z-axis (MPa)
l	defect length (mm)
w	defect width (mm)
d	defect depth (mm)
GA−ELM	extreme learning machine optimized by genetic algorithm
n	number of input layers
L	number of hidden layer neurons
$a_{i}$,$b_{i}$	hidden layer node parameters
$\beta_{i}$	outer weight between the i-th hidden layer node and the network output
g	activation function
H	hidden layer output matrix
J	squared loss function
N	determine population size
$P_{c}$	cross probability
$P_{m}$	mutation probability
$X\left(0\right)$	initial population
k	current evolutionary algebra
Y	maximum evolutionary algebra
h	safe allowable wall thickness of the pipe wall (m)
ρ	density of X100 steel (kg/m^3^)
T	erosion life (s)
